# Draft Genome Sequence of an *Enterococcus faecalis* Strain Isolated from a Neonatal Blood Sepsis Patient

**DOI:** 10.1128/genomeA.00907-14

**Published:** 2014-09-11

**Authors:** K. A. Kropp, A. Lucid, J. Carroll, V. Belgrudov, P. Walsh, B. Kelly, C. Smith, P. Dickinson, A. O’Driscoll, K. Templeton, P. Ghazal, R. D. Sleator

**Affiliations:** a Department of Biological Sciences, Cork Institute of Technology, Bishopstown, Cork, Ireland; b NSilico Lifescience Ltd., Bishopstown, Cork, Ireland; c Department of Computing, Cork Institute of Technology, Bishopstown, Cork, Ireland; d Division of Pathway Medicine, University of Edinburgh, Edinburgh, Scotland, United Kingdom; e Microbiological Diagnostic Unit, Royal Infirmary, University of Edinburgh, Edinburgh, Scotland, United Kingdom

## Abstract

Herein, we report the draft genome sequence of *Enterococcus faecalis* ED-NGS-1009, cultivated from a blood sample taken from a neonatal sepsis patient at the Royal Infirmary in Edinburgh, Scotland, United Kingdom.

## GENOME ANNOUNCEMENT

*Enterococcus faecalis* is a nosocomial, opportunistic pathogen showing resistance to antibiotics, specifically vancomycin (1–4). Preterm neonates are a highly susceptible patient group for bacterial infections (1, 5, 6) and rapid detection of blood sepsis and the causative pathogen are critical steps to enable proper treatment (7–9). Therefore, in the ClouDx-i project, we are aiming to extend our existing knowledge of currently circulating pathogenic strains linked with blood sepsis in neonates to inform the development of new molecular diagnostic assays. Herein, we present the draft genome sequence of an *Enterococcus faecalis* strain isolated from a preterm neonate at the Royal Infirmary in Edinburgh, in 2013. Positivity for blood sepsis and species identification were confirmed by classical microbiological techniques.

The isolate was grown overnight at 37°C on Luria broth (LB) agar, and genomic DNA was isolated using Qiagen genomic tips (Venlo, Limburg, Netherlands). Genomic DNA was fragmented (fragments 2 to 10 kb) using sonication and a non–size selected genome library was produced using the Nextera mate pair kit (Illumina, San Diego, CA). This library was then sequenced on an Illumina MiSeq using MiSeq Reagent kit version 3. Genomic sequence assembly, analysis, and automated reporting were carried out using Simplicity (10). This approach produced 2,954,539 total reads, resulting in an average 222-fold coverage. The average G+C content was 37.27%. Sequence assembly was achieved using a *de novo* assembly pipeline based on the SPAdes version 3.10 assembly tool with k-mers K21, K33, K55, K77, K99, and K127, resulting in a total of 79 contigs, of which 13 were >1,000 bp, representing 99.02% of the total sequence information with the largest contig being 2,285,020 bp in size. Postassembly processing was performed using SPAdes, and only scaffolds of >1,000 bp were considered when estimating genome size as 2,973,146 bp. We annotated the genome with Prokka (11) and used the identified 16S rRNA gene to confirm the species as *Enterococcus faecalis*. A scaffold of the genome was produced with Contiguator2 and we identified the closest related strain by BLASTing the scaffold, returning *E. faecalis* V583 and DENG1 as similar but not identical isolates, as was evident through insertions and deletions in the genome. The genome was then screened using GLIMMER3 (12) identifying 2,852 open reading frames (ORFs). The predicted ORFs were compared to the UniProt-TrEMBL database (13) using BLASTp, mapping 1,650 ORFs. To identify potential virulence factors in the genome, it was compared to a local database built from the VFDB (14) and Victors databases with BLASTp, using a 75% amino-acid sequence identity cutoff while only considering alignments longer than 100 amino-acids. In all, 63 potential virulence factors were identified.

Samples were handled in accordance with local ethical approval by the ethics committees of the NHS Lothian SAHSC Bioresource and the NHS Research and Development Office, Project ID 2011/R/NE/01 and the HSS BioResource Request ID 13/ES/0126.

### Nucleotide sequence accession numbers.

This whole-genome shotgun project has been deposited at DDBJ/EMBL/GenBank under the accession number JPWN00000000. The version described in this paper is version JPWN01000000.
